# *N*′-[(*E*)-1-(3-Fluoro­phen­yl)ethyl­idene]formohydrazide

**DOI:** 10.1107/S1600536809042809

**Published:** 2009-10-23

**Authors:** Zahid Shafiq, Muhammad Yaqub, M. Nawaz Tahir, Mian Hasnain Nawaz, M. Saeed Iqbal

**Affiliations:** a Department of Chemistry, Bahauddin Zakariya University, Multan 60800, Pakistan; b Department of Physics, University of Sargodha, Sargodha, Pakistan; c Department of Chemistry, Government College University, Lahore, Pakistan

## Abstract

In the title compound, C_9_H_9_FN_2_O, the dihedral angle between the fluoro­benzene ring and the mean plane of the side chain is 15.59 (14)°. In the crystal, the mol­ecules form inversion dimers linked by pairs of N—H⋯O hydrogen bonds, resulting in *R*_2_^2^(8) loops. These dimers are reinforced by C—H⋯O inter­actions.

## Related literature

For related structures, see: Shafiq *et al.* (2009*a* ▶,*b* ▶). For graph-set notation, see: Bernstein *et al.* (1995 ▶).
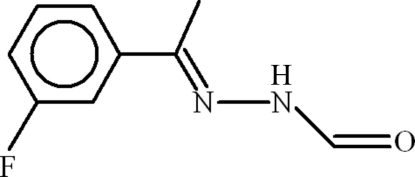


## Experimental

### 

#### Crystal data

C_9_H_9_FN_2_O
*M* *_r_* = 180.18
Triclinic, 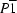

*a* = 6.8466 (5) Å
*b* = 7.0258 (6) Å
*c* = 9.9419 (8) Å
α = 70.558 (5)°
β = 81.267 (5)°
γ = 73.977 (4)°
*V* = 432.50 (6) Å^3^
*Z* = 2
Mo *K*α radiation
μ = 0.11 mm^−1^
*T* = 296 K
0.28 × 0.12 × 0.10 mm

#### Data collection

Bruker Kappa APEXII CCD diffractometer
Absorption correction: multi-scan (*SADABS*; Bruker, 2005 ▶) *T*_min_ = 0.986, *T*_max_ = 0.990
19438 measured reflections
2124 independent reflections
1320 reflections with *I* > 2σ(*I*)
*R*_int_ = 0.028

#### Refinement

*R*[*F* ^2^ > 2σ(*F* ^2^)] = 0.044
*wR*(*F* ^2^) = 0.148
*S* = 1.00
2124 reflections
119 parameters
H-atom parameters constrained
Δρ_max_ = 0.23 e Å^−3^
Δρ_min_ = −0.20 e Å^−3^

### 

Data collection: *APEX2* (Bruker, 2007 ▶); cell refinement: *SAINT* (Bruker, 2007 ▶); data reduction: *SAINT*; program(s) used to solve structure: *SHELXS97* (Sheldrick, 2008 ▶); program(s) used to refine structure: *SHELXL97* (Sheldrick, 2008 ▶); molecular graphics: *ORTEP-3* (Farrugia, 1997 ▶) and *PLATON* (Spek, 2009 ▶); software used to prepare material for publication: *WinGX* (Farrugia, 1999 ▶) and *PLATON*.

## Supplementary Material

Crystal structure: contains datablocks global, I. DOI: 10.1107/S1600536809042809/hb5150sup1.cif

Structure factors: contains datablocks I. DOI: 10.1107/S1600536809042809/hb5150Isup2.hkl

Additional supplementary materials:  crystallographic information; 3D view; checkCIF report

## Figures and Tables

**Table 1:** Hydrogen-bond geometry (Å, °)

*D*—H⋯*A*	*D*—H	H⋯*A*	*D*⋯*A*	*D*—H⋯*A*
N2—H2*A*⋯O1^i^	0.86	2.14	2.989 (2)	168
C8—H8*A*⋯O1^i^	0.96	2.52	3.204 (3)	129

Symmetry code: (i) 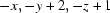.

**Fig. 1.**
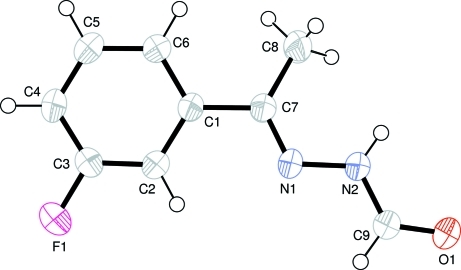
View of (I) with displacement ellipsoids drawn at the 50% probability level. H-atoms are shown by circles of arbitrary radius.

**Fig. 2.**
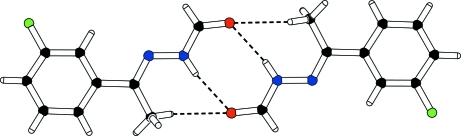
The partial packing of (I), which shows that molecules form dimers.

## supplementary crystallographic information

### Comment

Recently we have reported the crystal structures of (II) *N*'-[(1*E*)-1-(4-Chlorophenyl)ethylidene]formohydrazide (Shafiq *et al.*, 2009*a*), (III) *N*'-[(*E*)-(5-Methylfuran-2-yl)methylidene]formohydrazide (Shafiq *et al.*, 2009*b*). The title compound (I, Fig. 1) has been prepared in continuation of synthesizing various formohydrazide derivatives.

In (I), the groups A (C1—C6/F1) and B (C7/C8/N1/N2/C9) are planar with maximum r. m. s. deviations of 0.0022 and 0.0146 Å, respectively from their mean squares planes. The dihedral angle between A/B is 15.59 (14)°.

The molecules of (I) consist of dimers similar to (II) and (III) due to N–H···O type of intermolecular H-bondings forming *R*_2_^2^(8) ring motifs (Bernstein *et al.*, 1995). The difference between (I) and (II) is the substitution of Cl and F-atom on the *para* and *meta* positions of benzene ring, respectively. Due to this change there exist two *R*_2_^1^(7) ring motifs in dimers due to C–H···O and N—H···O H-bondings (Table 1).

### Experimental

To a hot stirred solution of formic hydrazide (1.0 g, 0.017 mol) in ethanol (15 ml) was added 1-(3-fluorophenyl)ethanone (2.043 ml, 0.017 mol). The resultant mixture was then heated under reflux. The reaction mixture was refluxed about 12 h and monitored through TLC. After the completion of reaction, the mixture was cooled to room temperature. The solid was collected by suction filtration. The product obtained was washed with hot ethanol and 1,4-dioxan and dried. Colourless needles of (I) were obtained by recrystallization of the crude product in 1,4-dioxan after two days.

### Refinement

The H-atoms were positioned geometrically (N—H = 0.86 Å, C—H = 0.93–0.96 Å) and refined as riding with *U*_iso_(H) = 1.2*U*_eq_(carrier) or 1.5*U*_eq_(methyl C).

### Crystal data

**Table:**

C_9_H_9_FN_2_O	*Z* = 2
*M**_r_* = 180.18	*F*(000) = 188
Triclinic, *P*1	*D*_x_ = 1.384 Mg m^−^^3^
Hall symbol: -P 1	Mo *K*α radiation, λ = 0.71073 Å
*a* = 6.8466 (5) Å	Cell parameters from 2124 reflections
*b* = 7.0258 (6) Å	θ = 3.1–28.3°
*c* = 9.9419 (8) Å	µ = 0.11 mm^−^^1^
α = 70.558 (5)°	*T* = 296 K
β = 81.267 (5)°	Cut needle, colourless
γ = 73.977 (4)°	0.28 × 0.12 × 0.10 mm
*V* = 432.50 (6) Å^3^	

### Data collection

**Table:**

Bruker Kappa APEXII CCD diffractometer	2124 independent reflections
Radiation source: fine-focus sealed tube	1320 reflections with *I* > 2σ(*I*)
graphite	*R*_int_ = 0.028
Detector resolution: 7.40 pixels mm^-1^	θ_max_ = 28.3°, θ_min_ = 3.1°
ω scans	*h* = −9→9
Absorption correction: multi-scan (*SADABS*; Bruker, 2005)	*k* = −9→9
*T*_min_ = 0.986, *T*_max_ = 0.990	*l* = −13→12
19438 measured reflections	

### Refinement

**Table:**

Refinement on *F*^2^	Primary atom site location: structure-invariant direct methods
Least-squares matrix: full	Secondary atom site location: difference Fourier map
*R*[*F*^2^ > 2σ(*F*^2^)] = 0.044	Hydrogen site location: inferred from neighbouring sites
*wR*(*F*^2^) = 0.148	H-atom parameters constrained
*S* = 1.00	*w* = 1/[σ^2^(*F*_o_^2^) + (0.0721*P*)^2^ + 0.1041*P*] where *P* = (*F*_o_^2^ + 2*F*_c_^2^)/3
2124 reflections	(Δ/σ)_max_ < 0.001
119 parameters	Δρ_max_ = 0.23 e Å^−^^3^
0 restraints	Δρ_min_ = −0.20 e Å^−^^3^

### Special details

**Table:**

Geometry. Bond distances, angles *etc*. have been calculated using the rounded fractional coordinates. All su's are estimated from the variances of the (full) variance-covariance matrix. The cell e.s.d.'s are taken into account in the estimation of distances, angles and torsion angles
Refinement. Refinement of *F*^2^ against ALL reflections. The weighted *R*-factor *wR* and goodness of fit *S* are based on *F*^2^, conventional *R*-factors *R* are based on *F*, with *F* set to zero for negative *F*^2^. The threshold expression of *F*^2^ > σ(*F*^2^) is used only for calculating *R*-factors(gt) *etc*. and is not relevant to the choice of reflections for refinement. *R*-factors based on *F*^2^ are statistically about twice as large as those based on *F*, and *R*- factors based on ALL data will be even larger.

### Fractional atomic coordinates and isotropic or equivalent isotropic displacement parameters (Å^2^)

**Table:**

	*x*	*y*	*z*	*U*_iso_*/*U*_eq_	
F1	−0.17799 (17)	0.19897 (19)	0.11146 (13)	0.0654 (5)	
O1	−0.27158 (19)	1.0743 (2)	0.48303 (16)	0.0571 (5)	
N1	−0.0361 (2)	0.6861 (2)	0.32755 (15)	0.0397 (4)	
N2	−0.0531 (2)	0.8255 (2)	0.40149 (15)	0.0423 (5)	
C1	0.1524 (2)	0.4305 (3)	0.22398 (18)	0.0385 (5)	
C2	−0.0221 (3)	0.3804 (3)	0.20534 (18)	0.0414 (5)	
C3	−0.0068 (3)	0.2466 (3)	0.12916 (19)	0.0443 (6)	
C4	0.1727 (3)	0.1578 (3)	0.0688 (3)	0.0600 (8)	
C5	0.3447 (3)	0.2084 (4)	0.0867 (3)	0.0734 (10)	
C6	0.3367 (3)	0.3417 (3)	0.1637 (2)	0.0582 (7)	
C7	0.1417 (2)	0.5780 (3)	0.30417 (18)	0.0395 (5)	
C8	0.3324 (3)	0.5870 (4)	0.3542 (3)	0.0689 (8)	
C9	−0.2381 (3)	0.9408 (3)	0.4233 (2)	0.0462 (6)	
H2	−0.14795	0.43732	0.24433	0.0496*	
H2A	0.05215	0.83756	0.43242	0.0507*	
H4	0.17812	0.06689	0.01769	0.0720*	
H5	0.46934	0.15168	0.04599	0.0882*	
H6	0.45566	0.37230	0.17538	0.0698*	
H8A	0.30519	0.60432	0.44755	0.1034*	
H8B	0.38093	0.70226	0.28885	0.1034*	
H8C	0.43388	0.46005	0.35838	0.1034*	
H9	−0.34809	0.91844	0.39133	0.0554*	

### Atomic displacement parameters (Å^2^)

**Table:**

	*U*^11^	*U*^22^	*U*^33^	*U*^12^	*U*^13^	*U*^23^
F1	0.0472 (7)	0.0812 (9)	0.0917 (9)	−0.0205 (6)	−0.0071 (6)	−0.0522 (7)
O1	0.0416 (7)	0.0611 (9)	0.0844 (10)	−0.0063 (6)	0.0031 (6)	−0.0510 (8)
N1	0.0379 (7)	0.0397 (8)	0.0486 (8)	−0.0064 (6)	−0.0014 (6)	−0.0256 (7)
N2	0.0350 (7)	0.0458 (8)	0.0558 (9)	−0.0057 (6)	−0.0034 (6)	−0.0312 (7)
C1	0.0353 (8)	0.0386 (9)	0.0457 (10)	−0.0038 (7)	−0.0042 (7)	−0.0217 (8)
C2	0.0348 (8)	0.0458 (10)	0.0482 (10)	−0.0059 (7)	0.0000 (7)	−0.0247 (8)
C3	0.0392 (9)	0.0480 (10)	0.0543 (11)	−0.0110 (8)	−0.0071 (7)	−0.0248 (9)
C4	0.0485 (11)	0.0681 (13)	0.0841 (15)	−0.0068 (9)	−0.0018 (10)	−0.0570 (12)
C5	0.0407 (10)	0.0940 (18)	0.112 (2)	−0.0058 (10)	0.0056 (11)	−0.0791 (16)
C6	0.0332 (9)	0.0723 (14)	0.0886 (15)	−0.0071 (9)	−0.0009 (9)	−0.0558 (12)
C7	0.0357 (8)	0.0408 (9)	0.0470 (10)	−0.0046 (7)	−0.0058 (7)	−0.0224 (8)
C8	0.0423 (10)	0.0802 (15)	0.1084 (18)	0.0019 (10)	−0.0197 (11)	−0.0673 (14)
C9	0.0356 (9)	0.0491 (10)	0.0638 (12)	−0.0082 (7)	−0.0003 (8)	−0.0330 (9)

### Geometric parameters (Å, °)

**Table:**

F1—C3	1.355 (2)	C4—C5	1.372 (3)
O1—C9	1.223 (3)	C5—C6	1.379 (3)
N1—N2	1.380 (2)	C7—C8	1.490 (3)
N1—C7	1.278 (2)	C2—H2	0.9300
N2—C9	1.332 (3)	C4—H4	0.9300
N2—H2A	0.8600	C5—H5	0.9300
C1—C2	1.389 (3)	C6—H6	0.9300
C1—C7	1.485 (3)	C8—H8A	0.9600
C1—C6	1.388 (3)	C8—H8B	0.9600
C2—C3	1.365 (3)	C8—H8C	0.9600
C3—C4	1.364 (3)	C9—H9	0.9300
			
N2—N1—C7	117.88 (15)	O1—C9—N2	123.78 (19)
N1—N2—C9	117.74 (15)	C1—C2—H2	120.00
C9—N2—H2A	121.00	C3—C2—H2	120.00
N1—N2—H2A	121.00	C3—C4—H4	121.00
C6—C1—C7	120.83 (15)	C5—C4—H4	121.00
C2—C1—C7	120.92 (16)	C4—C5—H5	119.00
C2—C1—C6	118.24 (18)	C6—C5—H5	119.00
C1—C2—C3	119.28 (19)	C1—C6—H6	120.00
C2—C3—C4	123.5 (2)	C5—C6—H6	120.00
F1—C3—C2	118.80 (18)	C7—C8—H8A	109.00
F1—C3—C4	117.74 (18)	C7—C8—H8B	109.00
C3—C4—C5	117.2 (2)	C7—C8—H8C	109.00
C4—C5—C6	121.4 (2)	H8A—C8—H8B	110.00
C1—C6—C5	120.5 (2)	H8A—C8—H8C	109.00
N1—C7—C1	115.92 (14)	H8B—C8—H8C	109.00
N1—C7—C8	124.86 (19)	O1—C9—H9	118.00
C1—C7—C8	119.20 (17)	N2—C9—H9	118.00
			
C7—N1—N2—C9	178.74 (16)	C2—C1—C7—C8	164.42 (19)
N2—N1—C7—C1	−179.88 (14)	C6—C1—C7—N1	164.85 (17)
N2—N1—C7—C8	1.7 (3)	C6—C1—C7—C8	−16.6 (3)
N1—N2—C9—O1	−177.46 (17)	C1—C2—C3—F1	−179.92 (16)
C6—C1—C2—C3	0.1 (3)	C1—C2—C3—C4	−0.2 (3)
C7—C1—C2—C3	179.02 (17)	F1—C3—C4—C5	179.6 (2)
C2—C1—C6—C5	0.4 (3)	C2—C3—C4—C5	−0.2 (4)
C7—C1—C6—C5	−178.6 (2)	C3—C4—C5—C6	0.7 (4)
C2—C1—C7—N1	−14.1 (3)	C4—C5—C6—C1	−0.8 (4)

### Hydrogen-bond geometry (Å, °)

**Table:**

*D*—H···*A*	*D*—H	H···*A*	*D*···*A*	*D*—H···*A*
N2—H2A···O1^i^	0.86	2.14	2.989 (2)	168
C8—H8A···O1^i^	0.96	2.52	3.204 (3)	129

Symmetry codes: (i) −*x*, −*y*+2, −*z*+1.
